# Provision of primary care pharmacy operated by hospital pharmacist

**DOI:** 10.3934/publichealth.2023020

**Published:** 2023-04-24

**Authors:** Suratchada Chanasopon, Kritsanee Saramunee, Tanupat Rotjanawanitsalee, Natt Jitsanguansuk, Surasak Chaiyasong

**Affiliations:** Social Pharmacy Research Unit, Faculty of Pharmacy, Mahasarakham University, Thailand 44150

**Keywords:** hospital pharmacy service, primary care pharmacy, primary care unit, WHO building blocks, Thailand

## Abstract

A primary care pharmacy (PCP) is operated by hospital pharmacists in Thailand. This study aims to explore the level of PCP provisions operated by hospital pharmacists, to identify health service components that affect PCP operation and to collect opinions from pharmacists regarding factors influencing PCP operation. A postal survey was conducted in northeastern Thailand. A questionnaire included: (1) the PCP checklist (36 items), (2) questions investigating the health service components required for PCP operation (13 items), and (3) queries to pharmacists concerning factors influencing PCP operation (16 items). Questionnaires were mailed to 262 PCP pharmacists. The PCP provision score was calculated with a max score of 36, and reaching at least 28.8 points was deemed as having ‘met expectation’. Multivariate logistic regression with a backward approach was used to determine health service components which affected PCP operation. Most respondents were female (72, 60.0%), aged 36.0 years (IQR 31.0–41.0) and PCP work experience of 4.0 years (IQR 2.0–10.0). Overall, the PCP provision score had met expectation (median = 29.00, Q_1_–Q_3_ = 26.50–32.00). Tasks that met expectation involved managing the medicine supply, a home visit with a multidisciplinary team and protecting consumer health. Improving medicine dispensary and promotion of self-care and herbal use were below expectation. PCP operation depended on doctor involvement (*OR* = 5.63 95% *CI* 1.07–29.49) and public health practitioner involvement (*OR* = 3.12 95% *CI* 1.27–7.69). The pharmacist's responsibility, i.e., a good relationship with the community, likely increased PCP provision. The PCP has been widely instituted in Northeast Thailand. Doctors and public health practitioners should get involved regularly. Further research is needed to monitor the outcomes and value of PCPs.

## 1. Introduction

The importance of primary health care has been increasingly recognized since the World Health Organization (WHO) published its 2008 health report, which indicated that primary health care can play a significant role in resolving health problems for people [1]. While Thailand has made huge investments in building up secondary and tertiary care in the past few decades, primary care has not been adequately supported [2],[3]. The Thai Ministry of Public Health has identified this as an important issue and announced a strategic plan for 2016–2026 that aims to strengthen primary healthcare services [3].

A primary care pharmacy (PCP) refers to pharmacists working with local healthcare professionals to maximize benefits and minimize the risk associated with medicines [4]. Community pharmacists have already been acknowledged for their contributions to primary care globally [5]. Pharmacists, however, work in other primary care settings, such as general practices, health centers and clinics. Research related to pharmacist roles in these primary care settings is scarce [6].

Thai primary healthcare is typically managed by a contracting unit for primary care, where a provincial or district hospital is the official commissioner contracted by the National Health Security Office. This means that the provincial or district hospital is the focal point of a network, mandating and controlling delivery of primary care services carried out by a primary care unit, referred to as a sub-district health promotion hospital (SHPH). An SHPH is a local health facility that delivers a range of public health services for people living in remote, rural areas [7]. To ensure accessibility to medicine at an SHPH, each SHPH is allowed to maintain a small stock of essential medicines. In addition, to achieve more seamless health care, an appropriate referral system must be established within the primary care network to permit rapid transfer of patients from the SHPH to facilities where higher levels of care can be provided when necessary [7],[8].

The promotion of primary healthcare policy has awoken all health professionals, including pharmacists. Since the Thai national pharmacy organizations have agreed that pharmacists should be involved in this important service, recent guidelines emphasize the role of the pharmacist as a critical component of the optimal delivery of primary care. The roles are referred to as ‘primary care pharmacy’ (PCP) and operated by hospital pharmacists [8],[9]. The PCP encompasses five tasks, which, together, aim to promote the safe use of medicines and other health products. The associated actions target the individual, the family and the community. Some of these tasks are performed at the level of the SHPH, while others are managed at the district level (Table 1).

Since the implementation of the PCP in 2010, all hospitals have attempted to perform these tasks. The mechanisms utilized to conduct these tasks has tended to differ from place to place depending on resources and health problems specific to each locality. Only two recently published reports have attempted to observe and assess the overall PCP provision in Thailand. The first study, conducted in Central Thailand, noted that the activity associated with managing the medicine supply was widely implemented in that area, while the other four PCP tasks were employed only moderately [10]. The second study was conducted in Southern Thailand; here, the authors reported a low level of success implementing most of the PCP tasks [11]. Importantly, a number of barriers were similarly highlighted in both reports. Investigators cited issues such as a shortage of staff and available technologies, inadequate remuneration and limitations in the pharmacists' time and competency to deliver primary care [10],[11]. However, there are also several reports describing the benefits of patient home visits by the primary care pharmacist. These visits were found to be valuable for resolving drug-related problems in chronic disease patients [12],[13], and for controlling glycemic levels in patients with diabetes [14]. Nonetheless, there has been a need for a research study where all PCP tasks are concurrently evaluated, in order to provide a simultaneous, comparative assessment across all tasks.

**Table 1.** Definition and goal of primary care pharmacy (PCP) tasks.

PCP tasks	Definition	Setting	Goal^a^
Managing medicine supply	Pharmacist must ensure accessibility to essential medicines in the SHPH by creating a list of essential medicines, supporting an appropriate procurement process and ensuring high-quality storage for medicine.	SHPH	At least 80% of SHPHs in the district area meet this standard.
Improving medicine dispensary service	Pharmacist must attempt to improve dispensing service in the SHPH by adhering to the 6R rules of dispensing medicine (right patient, right drug, right dose, right route, right technique and right time). All drugs should be carefully packaged and accurately labeled. Additional information for special patients should be available (e.g., pregnant patients, patients using warfarin, etc.)	SHPH	At least 80% of SHPHs in the district area meet this standard.
Home visit with multidisciplinary team	Pharmacist must provide pharmaceutical care as part of a multidisciplinary team at patients' homes. This service is offered to patients with chronic conditions such as mental health problems and chronic kidney disease, but it also includes palliative care and care for geriatric patients.	District	Home visit service by pharmacist and multidisciplinary team is available for patients who are in need of home care.
Consumer health protection	Pharmacist provides guidance for safe use of medicines and reports adverse events that may occur following use of any medicine or health care product by patients in the community.	District	Pharmacist performs this activity at least once a year.
Promoting self-care and herbal use	Pharmacist must encourage the public to embrace self-care for minor ailments and urge them to use over-the-counter medicines and herbal medicines commonly found in the community.	SHPH	At least 80% of SHPHs in district area have a campaign to promote this issue.

^a^Note: Followed national guidance of PCP assessment. Eighty percent is a criterion for indicating good compliance to primary care service delivery.

An exploration of the current status of facilities supporting the PCP is also clearly necessary. This will inform policy-makers as to which facilities are in greatest need of support. A 2010 publication by the WHO describing the essential building blocks for creating a strong health system framework has proven very useful for evaluating the status of health service support facilities [15]. Previous survey studies in India [16],[17], Ethiopia [18] and Moldova [19] explored the overall performance of health service delivery by evaluating supportive resources based on the WHO framework. While researchers in Zambia, Libya and Vietnam used this framework with a qualitative approach and mixed methods to observe how health service was delivered. Their results clearly identified facilities where primary care [16],[17],[19],[20] and hospital services [18] were in need of significant improvement. Therefore, based on published evidence, the building blocks of health systems described in the WHO report are considered a good framework for identifying resources required for optimal PCP operation. This study aims to explore the level of PCP provision operated by hospital pharmacists to identify health service components affecting PCP operation, and to collect opinions from pharmacists regarding factors influencing PCP operation.

## 2. Materials and methods

### 2.1. Study design

A cross-sectional study was conducted in Northeast Thailand from February 1 to May 31, 2017. Questionnaires were posted to the head of the pharmacy department of all community hospitals in this region (*n* = 262), specifying that the hospital pharmacist responsible for operating the PCP should complete the survey within one month. A stamped addressed envelope was provided. After the initial posting date, telephone reminders were made twice to the pharmacy department, at weeks 4 and 6.

### 2.2. Questionnaire

In order to achieve the study objectives, a questionnaire was generated to examine three aspects: (1) a checklist of PCP tasks, (2) health service components supporting service delivery based on the WHO framework of essential building blocks for health systems and (3) queries to pharmacists concerning factors influencing PCP operation.

#### 2.2.1. Provision of PCP

In Thailand, as mentioned previously, a hospital pharmacist is officially assigned the role of operating a PCP. The PCP is composed of five tasks (described in Table 1) that cover all levels of care, i.e., individual, family and community [8]. Next, a list of PCP activities within each task was drafted in the following manner. PCP activities created by the individual provincial public health offices were reviewed. This step was intended to allow researchers to learn about PCP activities used by the local public health office. Thirty-six activities were considered to be included in the questionnaire, a checklist of PCP tasks and activities, as shown in Supplement I.

#### 2.2.2. Building blocks of health systems

The “building blocks of a health system” refers to a framework recommended by the WHO whereby six salient components of a strong health system are defined. The definition of each component is briefly described elsewhere [15]. The questionnaire used in the current study was intended to explore components that are supportive of the PCP operation that constitute the six building blocks of a health system. The questionnaire addressed a range of issues that could reveal what and how PCP operation was supported, as shown in Table 2 (13 items). Four-point Likert scale statements (16 items) that sought the opinions of primary care pharmacists regarding factors that could support PCP operation were included in the last part of the questionnaire (Supplement II).

**Table 2.** Questions used to assess the building blocks of a health system.

Building block components	Questions
Service delivery	Does your family care team provide regular home visits to patients? Does your local government coordinate with you to deliver PCP service?
Health workforce	What type of health workforce is included in your family care team? (Doctor, nurse, pharmacist, public health practitioner, support staff) Did a pharmacist who is responsible for a PCP obtain appropriate training prior to commencing with this service?
Health information system	What type of patient database do you use? (electronic-based or paper-based; list all) Do you provide any type of educational media/materials for your patients? (leaflet/flyer, video, poster, etc.)
Access to essential medicines	What kind of vehicle do you use to provide services in the community? (car, van, motorcycle; list all) What kind of medical devices do you have to provide services in the community? (sphygmomanometer, glucose test device; list all)
Health system financing	Do you receive any financial support for the PCP services offered? What is the budget for the PCP services offered? Where do you get this budget?
Governance and leadership	Does the hospital director have a policy to support PCP services? Does the pharmacy head have a policy to support PCP services?

#### 2.2.3. Questionnaire validation

The first version of the questionnaire was drafted by researchers. Content validity was assessed by three experienced primary care pharmacists (who did not participate in the administration of the final version of the survey). The first draft was reviewed and checked for the sufficiency, clarity and relevance of items. Researchers revised the first draft based on this feedback. The second version of the questionnaire was piloted by 15 primary care pharmacists to ensure feasibility for collecting data, and to perform a reliability test. Some amendments were made to this version, but none affected the content of the key questions. Reliability was tested with 16 items, concerning factors influencing PCP operation; it confirmed the reliability of this questionnaire, as evidenced by a Cronbach alpha coefficient of 0.88. All PCP activities were weighted equally because they are essential for PCP performance.

### 2.3. Data analyses

#### 2.3.1. Calculation of PCP provision score

PCP provision was derived from Part I of the questionnaire (36 PCP activities). Responses from respondents were scored using two methods that were based on the PCP task setting: the SHPH level or district level (see Table 1). For the SHPH level, a respondent was given one point if at least 80% of the SHPHs had followed each of the selected activities. For the district level, one point was assigned if each of the selected activities was conducted by the respondent. The maximum possible score for PCP provision was 36; reaching at least 28.8 points (80% of 36) was deemed as having ‘met expectation’. This calculation method followed the national guidance of service quality assessment.

#### 2.3.2. Statistical analyses

Descriptive statistics were used to present an overview of the general characteristics of the respondents, an evaluation of the PCP provision and the opinions of primary care pharmacists. Univariate logistic regression was used to screen for components that had the potential for influencing PCP operation. The dependent variable was PCP provision, with a binary outcome: ‘met expectation’ (≥28.8) or ‘below expectation’ (<28.8). Thirteen independent variables regarding the building blocks of a health system were used as predictors of the model. Independent variables associated (*p* ≤ 0.200) with PCP provision were selected for further multivariate logistic regression. A backward approach was used to determine the best model for predicting PCP operation. A significance level for removal from the model was *p* > 0.200 [21],[22]. An odds ratio (*OR*) and its 95% confidence interval (95% *CI*) were used. A *p*-value of < 0.05 was the cut-off point for statistical significance.

### 2.4. Ethics approval of research

This study was granted ethical approval from The Human Research Ethics Committee of Mahasarakham University (approval no. 009/2559). All respondents were provided a participation information sheet. Returning a completed questionnaire was a virtue of consent to survey participation.

## 3. Results

### 3.1. Respondents

There were 121 completed questionnaires returned, denoting a 46.2% response rate. The majority of respondents were female (72, 60.0%), with a median age of 36.0 years (Q_1_–Q_3_ = 31.0–41.0) and approximately 4.0 years' (Q_1_–Q_3_ = 2.0–10.0) experience working with PCP. Respondents reported a median time of working in a PCP as being 2.4 hours per week (Q_1_–Q_3_ = 1.0–6.0). Most of respondents were from hospitals with ≤60 beds (97, 81.5%), and with a median number of SHPHs of 10.0 (Q_1_–Q_3_ = 6.0–15.0).

### 3.2. PCP provision

The reported PCP provisions are shown in Table 3. Overall, PCP provision was likely to meet expectation (median score of 29.00, Q_1_–Q_3_ = 26.50–32.00). However, just over half of the respondents (59, 54.63%) achieved the expected score of 28.8. Results from individual tasks revealed differences. For example, PCP tasks were reported as having been well performed in the ‘Managing medicine supply’ and ‘Consumer health protection’ domains, with median scores exceeding the expected level. On the other hand, the ‘Improving medicine dispensary service’ and ‘Promoting self-care and herbal use’ domains obtained scores below the expected level. In the case of the ‘Home visit with multidisciplinary team’ domain, the median score hit the expected range but only about half of the respondents (67, 55.83%) met the expectation.

**Table 3.** Performance of PCP provision.

PCP tasks	PCP provision score				Met expectation	
	Total	Met expectation	Median	Q_1_–Q_3_	*n*	%
Managing medicine supply (*n* = 110)	10.0	8.0	9.0	9.0–10.0	97	88.2
Improving medicine dispensary service (*n* = 112)	12.0	9.6	8.0	6.0–10.0	37	33.0
Home visit with multidisciplinary team (*n* = 120)	6.0	4.8	5.0	4.3–5.5	67	55.8
Consumer health protection (*n* = 121)	5.0	4.0	5.0	5.0–5.0	114	94.2
Promoting self-care and herbal use (*n* = 114)	3.0	2.4	2.0	2.0–3.0	55	48.3
Overall performance (*n* = 108)	36.0	28.8	29.0	26.5–32.0	59	54.6

Note: Q = Quartile.

### 3.3. Components supporting PCP services

Following the questions shown in Table 2, about two-thirds of respondents had conducted a regular home visit (80, 71.43%) with support from a local government (71, 63.96%). Most respondents reported having at least one doctor (95, 88.8%), at least one nurse (99, 94.3%) and at least one pharmacist (104, 97.2%) on a coordinated family care team. Of these, 64.6% of respondents reported that their pharmacists were trained in PCP. Just over half (60, 54.55%) stated that PCP services were financially supported, with funding coming directly from the governmental sector (58, 98.31%). A patient database system (electronic-based, paper-based or both) was likely to be established in most districts (83, 83.84%). Vehicles (118, 97.52%) and medical devices (106, 87.60%) were available to help deliver home care. PCP tasks were widely supported by hospital supervisors.

Univariate logistic regression indicated that several components were more likely to garner a ‘met expectation’ PCP provision score. However, multivariate logistic regression also confirmed that two independent variables significantly contributed to PCP operation. These included having at least one doctor (*OR* = 5.63 95% *CI* 1.07–29.49) and at least one public health practitioner (*OR* = 3.12 95% *CI* 1.27–7.69) on the family care team (Table 4).

### 3.4. Primary care pharmacists' perspective toward components supporting PCP operation

Most respondents tended to agree that the success of PCP operation was highly dependent on the pharmacist's responsibility in job assignment, a good relationship between the pharmacist and community and support being available to the pharmacist from supervisors (Table 5).

## 4. Discussion

Nowadays, pharmacists worldwide play an important role as healthcare professionals involved in primary care, especially in developed countries where community pharmacists can deliver a range of professional services [5],[23],[24]. PCP in Thailand is mainly delivered by a hospital pharmacist of the governmental sector [9]. Recent evidence also demonstrated the possibility of hospital pharmacists to deliver a home medication use review in Jordan [25]. Overall, the provision of PCP in Northeast Thailand had met expectation, with only a little over half of the survey participants (54.63%) reached the expected score. Respondents reported that they frequently performed in terms of managing supplies of medicines and promoting consumer health protection (>80% met expected score). The provision scores in these two domains are high because Thai hospital pharmacists have previously been assigned roles in these two domains and performed these duties for a long period of time prior to participating in the PCP. By contrast, the provision score of the pharmacist providing home visits to patients as a member of a multidisciplinary team was moderate. It is believed that this service requires strong collaboration among the members of the multidisciplinary team. This current study also confirms that the involvement of other health professionals in delivery of care has a significant impact on the high score of PCP provision. Nevertheless, Supper et al. found that pharmacists may be lacking training in how to work effectively with other professionals [26]. While awaiting to attend a PCP training course, it is suggested that pharmacists should regularly attend professional events in order to build up and maintain rapport with future team members from other disciplines [27]. Another task where respondents reported a below expectation of PCP provision was in the promotion of self-care and herbal use. This was due to pharmacists' limited knowledge of herbal medicines, as reported previously [28]. The area of weakest provision was the medicine dispensary service. The findings reported here differ from previous studies that were conducted in other regions of Thailand. An action research study conducted in a central province in 2013 reported that the only task to achieve a rating indicating a high level of provision was medicine supply management, while the remaining PCP domains were delivered with moderate satisfaction [10]. Another mixed-methods research study carried out in a southern province reported moderate to low performance for most domains [11]. Inconsistent findings between regions could reflect differences in questionnaires used by each study, as well as dissimilarities in the ways that PCP has been delivered in a different region.

The success of health service delivery is very dependent on a number of distinct components. According to the WHO recommendation, the six building blocks of health systems are the key elements that drive the health service system [15]. Therefore, all health professionals and stakeholders need to consider whether all components have been prepared adequately prior to the delivery of services. The current study illustrates that the PCP in Northeast Thailand had been effectively driven by the essential components of a successful health system, in accordance with the WHO's criteria. Still, such services too often lack several components including local collaboration, a public health practitioner, and educational materials, which limit to a full level of performance with health services. Particularly, as has been found using multivariate analysis, a competent health workforce (doctor and public health practitioner) in the family care team was significantly associated with a higher PCP provision score. One qualitative study previously conducted in Zambia identified a number of the barriers to delivery of health services, with the authors explaining that three critical components, along with many others, have affected the quality of health services in that country [29]. Generally, a doctor is recognized as a trustworthy health professional, since a previous survey reported that people would use public health services in community pharmacy if the doctor has advised them to do so [30]. Regarding components believed to have a significant impact on the high score of PCP provision from the perspective of respondents (pharmacists who are engaged in PCP delivery), the consensus was that the success of PCP operation was highly dependent on the responsibility and a good relationship with community of the pharmacist, the perceived knowledge and expertise of the pharmacist, and the extent of support given by hospital supervisors. Personality and other character traits are important components of healthcare provider competency, as they frequently determine how a person will behave or respond to others in certain situations [31]. Optimizing the amount of time available for the primary care pharmacist to work to provide service is another area that needs improvement. This survey established that, on average, pharmacists spent less than 3 hours per week delivering PCP services, which is less than the standard requirement [8].

**Table 4.** Health-service components supporting PCP operation.

Components supporting PCP operation^a^	*n* (%)	Crude *OR* (95% *CI*)	Adjusted *OR* (95% *CI*)
Service delivery			
Provide a regular home visit (*n* = 112)	80 (71.4)	2.84 (1.18–6.83)*	–
Coordination of local government (*n* = 111)	71 (63.9)	2.76 (1.21–6.32)*	–
Health workforce in family care team			
At least one doctor (*n* = 107)	95 (88.8)	6.75 (1.37–33.07)*	5.63 (1.07–29.49)**
At least one nurse (*n* = 105)	99 (94.3)	2.54 (0.44–14.54)	–
At least one pharmacist (*n* = 107)	104 (97.2)	2.56 (0.22–29.16)	–
At least one public health practitioner (*n* = 104)	61 (58.7)	2.42 (1.06–5.51)*	3.12 (1.27–7.69)**
Health information system			
Patient database (*n* = 94)	94 (100.0)	–	–
Educational media/materials (*n* = 111)	71 (63.9)	2.15 (0.95–4.88)	–
Access to essential medicines			
Vehicle (*n* = 115)	112 (97.39)	1.21 (0.74–19.91)	–
Medical device (*n* = 107)	92 (85.98)	2.27 (0.68–7.50)	–
Health system financing			
Obtain financial support (*n* = 110)	60 (54.6)	1.22 (0.56–2.68)	–
Annual budget^b^ = 20,000 baht (*IQR* = 40,000)			
Governance and leadership			
Policy supported by hospital director (*n* = 114)	107 (93.7)	5.18 (0.55–48.01)	–
Policy supported by pharmacy head (*n* = 114)	113 (99.1)	–	–

Note: ^a^A dichotomous outcome of PCP operation was used: did and did not meet expectation. ^b^Value is reported as median (IQR), *p < 0.200, **p < 0.05.

### 4.1. Strengths and limitations

This survey was conducted in a region of Thailand where PCP operations are abundant; thus, the findings are expected to be useful for planning further health service activities and support. Although two previous studies [10],[11] had already reported on levels of PCP provision, the studies were conducted within one province but focused on different aspects. Chalongsuk et al. explored an overview of information on how the PCP was managed and delivered [10]. Mateeapiruk observed PCP provision, but the questionnaire details were significantly different, especially the PCP activities used [11]. This, again, emphasized the need for the current study. Generalizability may be limited. Factors contributing to PCP operation can be transferable to other regions and countries; the involvement of doctors and public health practitioners in delivering PCP should be promoted. This study was a self-completion survey which could possibly have resulted in an over-interpretation by respondents. Multivariate logistic regression revealed that a wide range of 95% confidence intervals might be due to the small sample size. Referring the questionnaire to the pharmacy head could induce effects in two ways: (1) a negative effect by not passing on the questionnaire to the PCP pharmacist, which caused a low response rate, or (2) a positive effect by dominating respondents when filling the questionnaire, which led to bias. The exclusion of the construct and criterion validation unwarranted the accuracy of the PCP operation scores. However, the operation scores were re-reviewed by the local pharmacists to confirm the correctness of the results. Notably, there is no evaluation of expired stock or near-expired stock.

**Table 5.** Primary care pharmacist's perspective on components supporting PCP operation.

Components supporting PCP operation	Number of agreement	
	*n*	%
Responsibility of pharmacist in job assignment	107	88.4
Good relationship between the pharmacist and community	105	86.8
Support from a hospital director	104	86.0
Perseverance and patience of the pharmacist	103	85.1
Support from a pharmacy head	103	85.1
Knowledge of pharmacist regarding home pharmaceutical care	102	84.3
Skills of pharmacist to coordinate with community	102	84.3
Policy directed by a hospital director	102	84.3
Knowledge of pharmacist regarding medicine dispensing	101	83.5
Policy directed by a pharmacy head	97	80.2
Knowledge of pharmacist regarding consumer health protection	95	78.5
Existence of a family care team	93	76.9
Knowledge of pharmacist regarding medicine supply management	85	70.3
Complimentary expressed by pharmacy head	75	66.0
Complimentary expressed by a hospital director	66	54.6
Knowledge of pharmacist regarding self-care and herbal use	65	53.7

## 5. Conclusions

PCP provisions have been widely instituted in Northeast Thailand with the support of their leaders and some essential assets. The success of PCP operation is associated with the inclusion of a doctor and public health practitioner on the family care team, and they should be encouraged to get involved more regularly. In addition, a pharmacist's responsibility and relationship with the community are good indicators of successful PCP delivery. Further research is needed to monitor the outcomes and value of PCP.
