# Supplementary material for: Provision of primary care pharmacy operated by hospital pharmacist
Source: AIMS Public Health. 2023 Apr 24;10(2):268–80. doi: 10.3934/publichealth.2023020 (PMC10251043; doi:10.3934/publichealth.2023020)
Supplement: Supplementary file 1 [file publichealth-10-02-020-s001.pdf]

Public Health

AIMS Public Health, 10(2): 268–280.
DOI: 10.3934/publichealth.2023020
Received: 22 December 2022
Revised: 10 April 2023
Accepted: 11 April 2023
Published: 24 April 2023

http://www.aimspress.com/journal/aimsph

*Research article*

# Provision of primary care pharmacy operated by hospital pharmacist

**Suratchada Chanasopon, Kritsanee Saramunee*, Tanupat Rotjanawanitsalee, Natt Jitsanguansuk and Surasak Chaiyasong**

Social Pharmacy Research Unit, Faculty of Pharmacy, Mahasarakham University, Thailand 44150

* **Correspondence:** Email: kritsanee.s@msu.ac.th; Tel: +66043754360; Fax: +66043754360.

## Supplement I. A checklist of primary care pharmacy activities.

**Managing medicine supply (Max possible score=10)**

***Question: Indicate the number of SHPHs which can provide the following activities***

1. There is a stock of medicine that is held in a secure location and separated from other work area.
2. There is a security system to protect medicine from theft.
3. The stock of medicine is clean with good control of storage conditions such as sunlight, humidity, and flooding.
4. The temperature of a stock of medicine should not exceed 30 °C.
5. Medicines and medical supplies are organised in a logical manner with clear labelling.
6. Storage of vaccines follows the 'cold chain system' rules.
7. Essential emergency drugs are available for use, including adrenaline injection 1:1000, atropine injection 1:1000, 0.5% calcium chloride/gluconate, 20% or 50% dextrose, furosemide injection, and hydrocortisone/dexamethasone.
8. Medicines and medical supplies are removed from the stock based on the "First Expire First Out (FEFO)" rule.
9. Inventory management is used to maintain appropriate quantities of stock.
10. There is a list of essential drugs that reflects the specific health needs of the local community.

**Improving medicine dispensary services (Max possible score=12)**

***Question: Indicate the number of SHPHs which can provide the following activities***

1. There is a system for monitoring medication errors.
2. A computerised system produces medicine labels with the patient's name and important information about the medicine.
3. Printed material describing appropriate use of the medicine is provided with the medicine (auxiliary labels, educational leaflet, etc.).

4. History of drug allergy is always recorded in a patient's profile.
5. A drug allergy card is issued to a patient with a history of allergic reactions.
6. A history of drug allergy is shared with a hospital.
7. If a patient is taking warfarin, pharmacists/health care staff are alerted to this by a signal in the patient's health record.
8. A sign with statement "Please inform pharmacist/health care staff if you are pregnant or breast feeding" is shown clearly in the dispensing area.
9. A sign with statement "Please inform staff if allergic to any medicine" is shown clearly in the dispensing area.
10. An alert sign is shown clearly on the shelf of drugs that are contraindicated during pregnancy.
11. A list of drugs contraindicated during pregnancy is available.
12. There is training for non-pharmacist health professionals regarding dispensing of medicine.

**Home visit with multidisciplinary team (Max possible score=6)**

***Question: Indicate whether the following activities are conducted in your district.***

1. You and your team know the exact number of patients who are in need of continuity of care such as chronic disease patients, palliative patients, etc.
2. You and your team provide home visits.
3. A case conference is held by the family care team to prepare and summarise a medical plan for the patient, before and after home visit.
4. Continuity of care is provided to at least 60% of patients who need it.
5. Medicine is delivered directly to a patient's home if they cannot visit a hospital.
6. A family care team always reports incidences of adverse drug reactions to the centre that monitors adverse product reactions.

**Consumer health protection (Max possible score=5)**

***Question: Indicate whether the following activities are conducted in your district.***

1. You and your team inspect local health businesses at least twice a year to monitor for possible health care violations.
2. You and your team inspect local businesses (restaurants, convenient stores, open markets, etc.) at least once a year to monitor for possible health care violations.
3. You and your team organise an educational event for consumer empowerment.
4. You and your team inspect local businesses that likely sells hazardous products to the community.
5. You and your team are proactive with regard to identifying and eliminating problems due to improper use of medicine and health products.

**Promoting self-care and herbal use (Max possible score=3)**

***Question: Indicate the number of SHPHs which can provide the following activities***

1. Community members are provided with information to educate them about the use of over-the-counter medicines.
2. There is a place/centre located in the community to support the use of over-the-counter medicines.
3. Herbal medicines endorsed in the National List of Herbal Medicine 2013 are recommended for use at home.

Note: SHPH: sub-district health promoting hospital which is a place for providing primary healthcare. There is no evaluation of expired stock or near-expired stock.

## Supplement II. Queries to pharmacists concerning factors influencing PCP operation (16 items).

| Items | Level of impact to PCP operation | | | |
|---|---|---|---|---|
| Responsibility of pharmacist in job assignment | ☐ high | ☐ moderate | ☐ low | ☐no impact |
| Good relationship between a pharmacist and community | ☐ high | ☐ moderate | ☐ low | ☐no impact |
| Support from a hospital director | ☐ high | ☐ moderate | ☐ low | ☐no impact |
| Perseverance and patience of a pharmacist | ☐ high | ☐ moderate | ☐ low | ☐no impact |
| Support from a pharmacy head | ☐ high | ☐ moderate | ☐ low | ☐no impact |
| Knowledge of pharmacist regarding home pharmaceutical care | ☐ high | ☐ moderate | ☐ low | ☐no impact |
| Skills of pharmacist to coordinate with community | ☐ high | ☐ moderate | ☐ low | ☐no impact |
| Policy directed by a hospital director | ☐ high | ☐ moderate | ☐ low | ☐no impact |
| Knowledge of pharmacist regarding medicine dispensing | ☐ high | ☐ moderate | ☐ low | ☐no impact |
| Policy directed by a pharmacy head | ☐ high | ☐ moderate | ☐ low | ☐no impact |
| Knowledge of pharmacist regarding consumer health protection | ☐ high | ☐ moderate | ☐ low | ☐no impact |
| Existence of a family care team | ☐ high | ☐ moderate | ☐ low | ☐no impact |
| Knowledge of pharmacist regarding medicine supply management | ☐ high | ☐ moderate | ☐ low | ☐no impact |
| Complimentary expressed by pharmacy head | ☐ high | ☐ moderate | ☐ low | ☐no impact |
| Complimentary expressed by a hospital director | ☐ high | ☐ moderate | ☐ low | ☐no impact |
| Knowledge of pharmacist regarding self-care and herbal use | ☐ high | ☐ moderate | ☐ low | ☐no impact |

AIMS Press

© 2023 the Author(s), licensee AIMS Press. This is an open access article distributed under the terms of the Creative Commons Attribution License (http://creativecommons.org/licenses/by/4.0)
